# Assessment of population susceptibility to upcoming seasonal influenza epidemic strain using interepidemic emerging influenza virus strains

**DOI:** 10.1017/S0950268819001717

**Published:** 2019-09-26

**Authors:** Lin-Lei Chen, Wai-Lan Wu, Wan-Mui Chan, Carol H. Y. Fong, Anthony C. K. Ng, Jonathan D. Ip, Lu Lu, Thrimendra K. Dissanayake, Xixia Ding, Jian-Piao Cai, Anna J. X. Zhang, Sidney Tam, Ivan F. N. Hung, Kwok-Hung Chan, Kwok-Yung Yuen, Kelvin K. W. To

**Affiliations:** 1 Department of Microbiology, Li Ka Shing Faculty of Medicine, The University of Hong Kong, Pokfulam, Hong Kong Special Administrative Region, China; 2 Laboratory of Emerging Infectious Diseases and Division of Laboratory Medicine, Zhujiang Hospital, Southern Medical University, Guangzhou, China; 3 Division of Clinical Biochemistry, Department of Pathology, Queen Mary Hospital, Hong Kong Special Administrative Region, China; 4 Department of Medicine, Li Ka Shing Faculty of Medicine, The University of Hong Kong, Pokfulam, Hong Kong Special Administrative Region, China; 5 State Key Laboratory for Emerging Infectious Diseases, Li Ka Shing Faculty of Medicine, The University of Hong Kong, Hong Kong Special Administrative Region, China; 6 Carol Yu Centre for Infection, The University of Hong Kong, Hong Kong Special Administrative Region, China; 7 Department of Clinical Microbiology and Infection Control, The University of Hong Kong-Shenzhen Hospital, Shenzhen, China

**Keywords:** Influenza, respiratory tract infections, serology, population susceptibility

## Abstract

Seasonal influenza virus epidemics have a major impact on healthcare systems. Data on population susceptibility to emerging influenza virus strains during the interepidemic period can guide planning for resource allocation of an upcoming influenza season. This study sought to assess the population susceptibility to representative emerging influenza virus strains collected during the interepidemic period. The microneutralisation antibody titers (MN titers) of a human serum panel against representative emerging influenza strains collected during the interepidemic period before the 2018/2019 winter influenza season (H1N1-inter and H3N2-inter) were compared with those against influenza strains representative of previous epidemics (H1N1-pre and H3N2-pre). A multifaceted approach, incorporating both genetic and antigenic data, was used in selecting these representative influenza virus strains for the MN assay. A significantly higher proportion of individuals had a ⩾four-fold reduction in MN titers between H1N1-inter and H1N1-pre than that between H3N2-inter and H3N2-pre (28.5% (127/445) *vs.* 4.9% (22/445), *P* < 0.001). The geometric mean titer (GMT) of H1N1-inter was significantly lower than that of H1N1-pre (381 (95% CI 339–428) *vs.* 713 (95% CI 641–792), *P* < 0.001), while there was no significant difference in the GMT between H3N2-inter and H3N2-pre. Since A(H1N1) predominated the 2018–2019 winter influenza epidemic, our results corroborated the epidemic subtype.

## Introduction

Seasonal influenza virus infection has been associated with an estimated 9.4 million respiratory hospitalisations and an estimated 0.3 to 0.6 million deaths per year globally [1, 2]. During influenza epidemics, the sudden surge in the number of patients attending out-patient clinics and hospitals leads to overcrowded clinics and hospital wards, and increased workload of healthcare workers [3, 4]. The total healthcare and society cost has been estimated to be US $11.2 billion per year in the United States [5].

Seasonal influenza epidemics are caused by influenza A(H1N1), A(H3N2) and influenza B virus. There are important epidemiological differences between these influenza viruses [6]. Studies have shown that the median ages of patients with influenza A(H1N1) (20 years) and influenza B (16 years) virus infection are younger than those with influenza A(H3N2) (30 years) virus infection [7]. For influenza B virus, patients infected by the Victoria lineage are younger than those infected with the Yamagata lineage (median age: 20 years *vs.* 40 years) [8]. Influenza A(H1N1) virus has also been associated with higher incidence of intensive care unit admission [6]. After the 2009 pandemic, the mortality rate was higher for A(H3N2) virus than A(H1N1) virus for older patients born before 1946, but was higher for A(H1N1) virus for younger patients born after 1947 [9]. Vaccine effectiveness is much lower for influenza A(H3N2) virus than influenza A(H1N1) or influenza B virus [10]. These differences in epidemiological characteristics and vaccine effectiveness have significant implication in healthcare resource and workforce planning for an influenza season.

An antibody titer against an influenza virus strain correlates with protection against antigenically similar strains [11]. As influenza virus strains evolve, the population antibody titer against the new strains may be reduced, and these new strains will emerge as the predominant strain [12]. For example, the influenza virus A(H1N1)pdm09 has quickly spread around the world because most people, except the elderly born near the 1918 pandemic, do not have protective antibody against the new virus [13–15].

The aim of this study is to determine the population susceptibility to influenza viruses that are newly emerging in the interepidemic period. We used a human serum panel consisting of individuals from all age groups as we described previously [12, 16]. We hypothesise that the influenza subtype with a greater reduction in the antibody titer would signify an increased susceptibility of the population to that subtype.

## Methods

### Patient samples

We screened 445 random anonymised archived serum samples from the clinical biochemistry laboratory of Queen Mary Hospital in Hong Kong as we described previously [12]. The serum samples consisted of 50 samples of each 10-year age cohort from 10–19 year-old to ⩾80 year-old cohorts. For the 0–9 year-old cohort, 45 serum samples were retrieved. These serum samples were collected from April to June 2018, which is after the 2017/2018 winter influenza season. This study was approved by the HKU/HA HKW Institutional Review Board (UW 18–141).

### Choosing influenza A virus strains for microneutralisation assay

Influenza A strains representative of previous epidemics (H1N1-pre and H3N2-pre) and those representative of emerging influenza strains collected during the interepidemic period before the 2018/2019 winter influenza season (H1N1-inter and H3N2-inter) were chosen based on genetic and antigenic data that are publicly available. These include the antigenic data published by the World Health Organisation [17], and the genetic information available at the Global Initiative on Sharing All Influenza Data (GISAID) [18]. The amino acid sequences were aligned using FAMSA [19]. The nucleotide sequences of A/HK/412489/2016, A/HK/439315/2018 and A/HK/417610/2018 have been deposited on the GISAID EpiFlu database under accession numbers EPI1331036-EPI1331038.

### Microneutralisation assay

A microneutralisation (MN) assay was performed and interpreted according to the 2-day enzyme-linked immunosorbent assay protocol of the World Health Organisation [20, 21]. Serum samples were serially diluted by two-fold from 1:20 to 1:2560. Viral antigen was detected using anti-nucleoprotein antibody [22]. All viruses used in the MN assay were cultured in Madin Darby canine kidney cells as we described previously [12], to avoid mutations that may arise during egg passage. The haemagglutinin (HA) gene of the virus stocks used for the MN assay was sequenced.

### Statistical analysis

Statistical analysis was performed using SPSS 23. For statistical analysis, a value of 2560 was assigned if the MN titer was ⩾2560. The McNemar test was used in comparing the proportion of serum specimens with ⩾four-fold reduction in the MN titer. The paired-sample *t* test was used in comparing the geometric mean titers (GMT). Log-transformed MN titers were used for the statistical analysis of the GMT and 95% confidence interval (CI) as we described previously [23, 24].

## Results

### Selection of influenza A(H1N1) strains

**Fig. 1.**
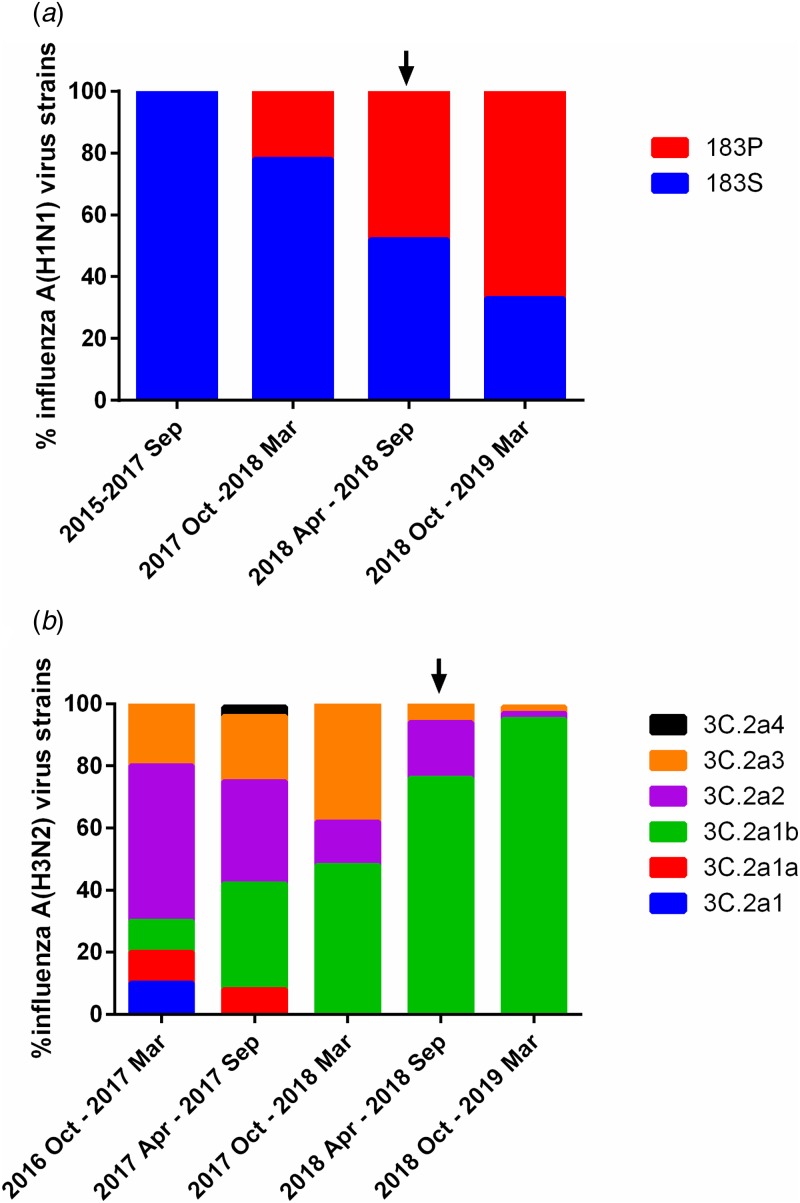
Influenza A strains emerging in Hong Kong. (a) Emergence of influenza A(H1N1) strains with HA S183P substitution. (b) Emergence of influenza A(H3N2) strains belonging to lineage 3C.2a1b. Amino acid sequences were downloaded from GISAID (Supplementary Table S1). Serum samples in this study were collected from April to June 2018 and are indicated by the black arrows.

Before the 2018/2019 winter influenza season, the last A(H1N1) epidemic occurred in the 2015/2016 winter influenza season. S183P substitution in the HA, which was absent in A(H1N1) virus strains collected between 2015 and September 2017, was increasingly found among A(H1N1) strains collected in Hong Kong (Fig. 1a). HA S183P was highlighted as a marker of emerging A(H1N1) virus strains according to the World Health Organisation [17]. Hence, for H1N1-pre, we have chosen a strain with HA 183S (A/HK/412489/2016). For H1N1-inter, we have chosen a strain with HA 183P (A/HK/439315/2018), which was isolated from an adult patient with severe disease requiring extracorporeal membrane oxygenation. No mutations in the HA gene were found during virus passage for both H1N1-pre and H1N1-inter.

### Selection of influenza A(H3N2) strains

**Fig. 2.**
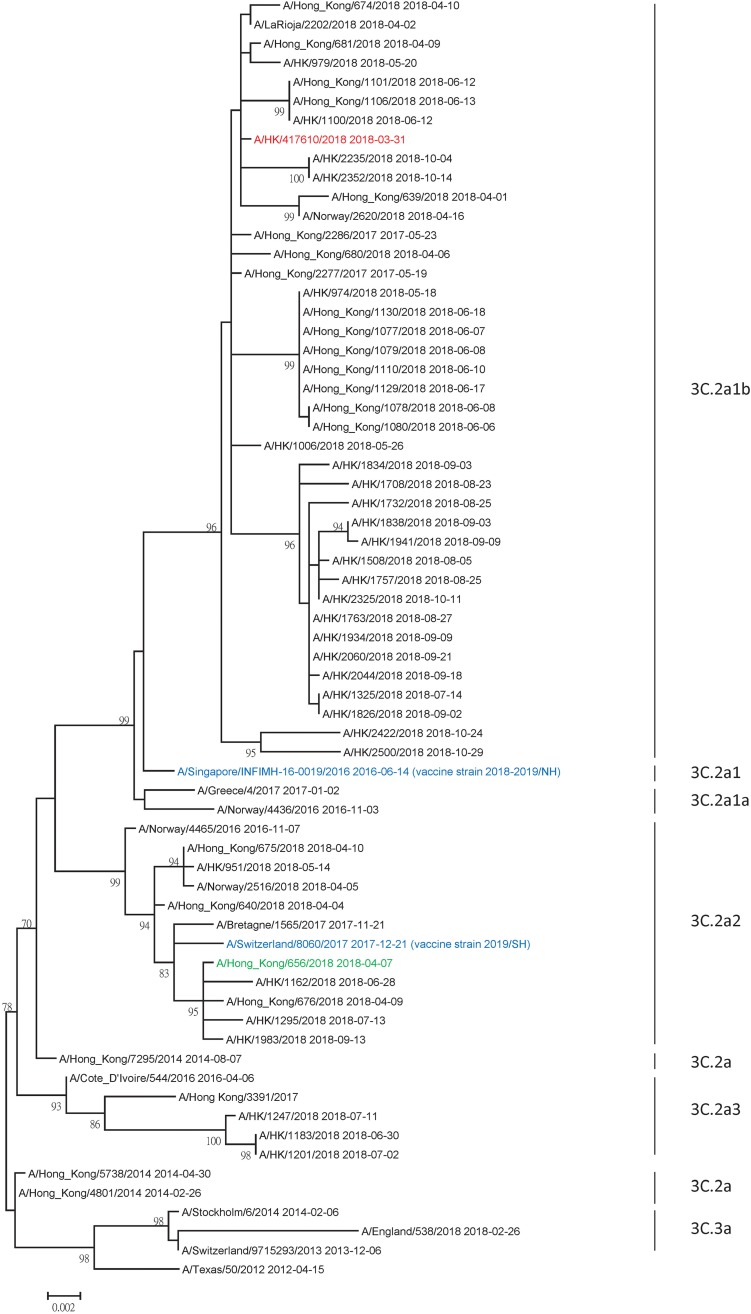
Phylogenetic tree of HA showing the genetic relationship of influenza A(H3N2) in Hong Kong. Nucleotide sequences were downloaded from GISAID (Supplementary Table S2). All influenza A(H3N2) strains from Hong Kong available at GISAID as of 7 January 2019 are included. Vaccine strains recommended by the World Health Organisation are highlighted in blue. H3N2-pre and H3N2-inter used in the MN assay are highlighted in green and red, respectively. The phylogenetic trees were constructed using the maximum-likelihood method with the best-fit substitution model HKY + G. Bootstrap values were calculated from 1000 trees.

Phylogenetic analysis showed that A(H3N2) clade 3C.2a2 and 3C.2a1b predominated in the 2017 summer epidemic in Hong Kong. However, only 3C.2a1b rapidly increased in 2018, accounting for 76% of the strains tested between April and September of 2018 (Fig. 1b). In the antigenic analysis by the World Health Organisation, A(H3N2) virus strains in the clade 3C.2a1b have different antigenic characteristic from strains in the clade 3C.2a2. Hence, for H3N2-pre, we have chosen a strain that belongs to clade 3C.2a2 (A/Hong Kong/656/2018; GISAID accession number EPI_ISL_312267). In the antigenic analysis by the World Health Organisation, A/Hong Kong/656/2018 is antigenically similar to egg-passaged A/Switzerland/8060/17, which is the recommended H3N2 vaccine strain [17]. For H3N2-inter, we have chosen a strain belonging to clade 3C.2a1b with 135K (A/HK/417610/2018) (Fig. 2). No mutations in the HA gene were found during virus passage for H3N2-pre. For H3N2-inter, one mutation (T160K) was found during passage.

### Comparison of MN titers between previous epidemic strain and emerging strain

The MN titers for H1N1-inter were ⩾four-fold or ⩾eight-fold lower than those of H1N1-pre for 28.5% (127/445) and 10.3% (46/445) of individuals, respectively (Table 1). In comparison, the MN titers for the H3N2-inter were ⩾four-fold or ⩾eight-fold lower than those for H3N2-pre for only 4.9% (22/445) and 1.1% (5/445), respectively. Overall, the proportion of individuals with ⩾four-fold or ⩾eight-fold reduction in MN titers between the previous epidemic strains and the interepidemic emerging strains was significantly higher for A(H1N1) than that of A(H3N2) (*P* < 0.001). Subgroup analysis also showed that the proportion of individuals with ⩾four-fold reduction in MN titers between the previous epidemic strains and the interepidemic emerging strains of A(H1N1) was higher than that of A(H3N2) for all nine different age groups. The difference is most striking for the 30–39 year-old age group, in which 58% of individuals had ⩾four-fold reduction in H1N1 titer *vs.* 2% for H3N2.

**Table 1.** Comparison of microneutralisation antibody titer between influenza A virus strains representative of previous epidemics and those emerging during the interepidemic period

	No. (%) with ⩾four-fold reduction in MN titer between strains representative of previous epidemics and those emerging during the interepidemic period			No. (%) with ⩾eight-fold reduction in MN titer between strains representative of previous epidemics and those emerging during the interepidemic period		
Age group (years)^a^	H1N1	H3N2	*P* value^b^	H1N1	H3N2	*P* value^b^
0–9	6 (13)	0 (0)	0.031	1 (2.2)	0 (0)	1.000
10–19	6 (12)	2 (4)	0.289	2 (4)	0 (0)	0.500
20–29	14 (28)	4 (8)	0.013	2 (4)	1 (2)	1.000
30–39	29 (58)	1 (2)	<0.001	14 (28)	0 (0)	<0.001
40–49	11 (22)	5 (10)	0.146	4 (8)	1 (2)	0.375
50–59	25 (50)	3 (6)	<0.001	13 (26)	0 (0)	<0.001
60–69	10 (20)	4 (8)	0.180	5 (10)	2 (4)	0.453
70–79	17 (34)	1 (2)	<0.001	5 (10)	0 (0)	0.063
⩾ 80	9 (18)	2 (4)	0.065	0 (0)	1 (2)	1.000
Total (*n* = 445)	127 (28.5)	22 (4.9)	<0.001	46 (10.3)	5 (1.1)	<0.001

a *n* = 50 in each age group, except *n* = 45 for 0–9 year-old age group.

b *P* value calculated using the McNemar test.

The GMT for H1N1-inter was significantly lower than that for H1N1-pre (381 (95% CI 339–428) *vs.* 713 (95% CI 641–792), *P* < 0.001). Conversely, there was no significant difference in the GMT between H3N2-pre and H3N2-inter (523 (95% CI 462–592) *vs.* 523 (95% CI 469–583), *P* = 1.000). The GMT for H1N1-inter was significantly lower than that for H1N1-pre for all age groups (Table 2). For H3N2, only the 20–29 year-old age group had lower GMT for the H3N2-inter than H3N2-pre.

**Table 2.** Geometric mean microneutralisation antibody titer against influenza A virus of each age group

	Geometric mean microneutralisation titer					
Age group (years)^a^	H1N1-pre	H1N1-inter	*P* value^b^	H3N2-pre	H3N2-inter	*P* value^b^
0–9	819 (565–1188)	621 (416–925)	0.004	470 (325–681)	630 (454–875)	<0.001
10–19	1512 (1213–1884)	1162 (887–1522)	0.005	1554 (1201–2012)	1372 (1087–1732)	0.071
20–29	696 (509–951)	383 (277–529)	<0.001	868 (616–1223)	686 (496–948)	0.020
30–39	715 (526–972)	220 (165–293)	<0.001	316 (225–442)	348 (262–462)	0.279
40–49	411 (296–570)	197 (148–263)	<0.001	334 (222–502)	320 (224–457)	0.690
50–59	549 (396–762)	194 (142–266)	<0.001	246 (185–328)	239 (190–302)	0.749
60–69	389 (277–545)	253 (179–357)	0.001	338 (229–499)	348 (256–474)	0.811
70–79	957 (739–1239)	513 (372–707)	<0.001	676 (484–946)	725 (525–1001)	0.471
⩾80	931 (700–1237)	589 (429–809)	<0.001	766 (522–1124)	745 (544–1021)	0.749
Total	713 (641–792)	381 (339–428)	<0.001	523 (462–592)	523 (469–583)	1.000

H1N1-inter, A(H1N1) interepidemic strain; H1N1-pre, A(H1N1) strain representative of previous epidemic.

Data are geometric mean microneutralisation titer (95% CI).

a *n* = 50 in each age group, except *n* = 45 for 0–9 year-old age group.

b *P* value calculated using the paired sample *t*-test with a log-transformed MN titer.

### Use of pooled serum specimens for comparing MN titers

The testing of MN titers of individual serum is labour intensive and time consuming. Hence, we determined whether serum specimens from different individuals can be pooled together for MN testing. From each age group, we have pooled serum specimens from all individuals and the MN titers against H1N1-pre and H1N1-inter were determined. The difference of MN titers between H1N1-pre and H1N1-inter for all age groups was within one dilution, except for the age group 60–69 for which the MN titer of H1N1-inter was four-fold lower than that of H1N1-pre (Table 3).

**Table 3.** Microneutralisation antibody titer of pooled serum against influenza A H1N1 virus of each age group

	Microneutralisation antibody titer		
Age group (years)^a^	H1N1-pre	H1N1-inter	H1N1-pre/H1N1-inter
0–9	1280	1280	1
10–19	2560	2560	1
20–29	1280	640	2
30–39	320	320	1
40–49	320	320	1
50–59	640	320	2
60–69	1280	320	4
70–79	1280	640	2
⩾80	1280	640	2

H1N1-inter, A(H1N1) interepidemic strain; H1N1-pre, A(H1N1) strain representative of previous epidemic.

a *n* = 50 in each age group, except *n* = 45 for 0–9 year-old age group.

## Discussion

Influenza virus causes seasonal epidemics worldwide every year, putting a significant burden on the healthcare system. Assessing the population susceptibility to the upcoming epidemic influenza strain is one of the important components in preparing for influenza epidemics. In this study, we determined the population susceptibility by comparing the antibody titers against representative influenza virus strains that emerge during the interepidemic period with influenza virus strains representative of those in the previous epidemic. From a human serum panel from 445 patients encompassing all age groups from <10 to ⩾80 years of age, 28.5% of individuals had ⩾four-fold lower MN titers against H1N1-inter compared with H1N1-pre, while only 4.9% had ⩾four-fold lower MN titers against H3N2-inter compared with H3N2-pre. For the influenza season 2018/19 winter influenza season in Hong Kong, A(H1N1) was the predominant subtype affecting Hong Kong, and the epidemic peak in the current season is much more severe than the A(H1N1) 2017/2018 winter or 2017 summer peak [25]. Similarly, A(H1N1) subtype affects most hospitalised patients with laboratory-confirmed influenza virus infection in the 2018/2019 season in Europe [26]. A(H1N1) is also the most predominant influenza virus subtype affecting the United States [27]. Therefore, the findings from our serosurveillance of interepidemic influenza virus strains, which were collected before the 2018/2019 winter epidemic, corroborated with the predominant influenza virus subtype in the 2018/2019 winter epidemic.

Although the predominant influenza virus strains of a particular influenza subtype during an influenza season can only be ascertained after the influenza season has begun, these can be predicted by analysing the influenza virus strains collected during the interepidemic period. As seen in Figure 1a, HA S183P substitution, which was found in most A(H1N1) strains collected in the 2018/19 winter influenza season, showed a clear trend of increase since the last A(H1N1) predominant season in 2015/16 winter. Our approach, which used representative emerging influenza strains collected during the interepidemic period, provides population susceptibility data before an epidemic has been started. The population susceptibility data that are available before an epidemic would guide resource allocation.

Our serum panel consists of individuals from all age groups. This is important because antibodies from individuals of different ages have different antiviral properties. Xie *et al*. have shown that antigenic distance determined using sera from children does not correlate with that determined using sera from adults [28].

Some studies have tested post-vaccination human serum with emerging strains [17]. This approach is useful in predicting vaccine effectiveness. However, the data is not useful in predicting population susceptibility to emerging strains in areas with a low-vaccine uptake rate. In Hong Kong, the overall seasonal influenza uptake rate was only 14.8% as of 3 March 2019 [29]. The vaccination rate for those not eligible in the government vaccination program, such as young healthy adults without chronic medical illness, is likely to be lower. Since A(H1N1) disproportionately affects the younger population, our study approach is particularly relevant.

Our result is in stark contrast with the results using post-infection ferret antisera. According to the World Health Organisation and European Centre for Disease Prevention and Control, there was no significant antigenic difference between old and circulating strains of A(H1N1) as determined by ferret antisera [17, 30]; however, there was a significant reduction in ferret serum neutralisation titer against the circulating A(H3N2) genetic clade 3C.2a1b when compared with that clade 3C.2a2 [17, 31]. Several reasons may account for the difference. First, our study uses human serum panel instead of ferret panel. Many studies have demonstrated that the results from human and ferret may be different. Second we use microneutralisation assay instead of HA inhibition assay. Traditionally, antigenic characteristics of influenza viruses are determined by haemagglutination inhibition assay (HAI) using post-infection ferret antisera [15, 32, 33], and antigenic distance can be derived from the difference in HAI between strains [34]. However, recent A(H3N2) strains poorly agglutinate red blood cells, and therefore HAI cannot be performed for these viruses [17, 30].

Other groups have developed models to predict the predominant influenza virus subtype in the upcoming influenza season. These models are based on the evolution rate, or specific mutations in the HA [35, 36]. The addition of serosurveillance data using emerging strains in the interepidemic period may strengthen these models.

Pooled serum panels have been used by some groups in determining the antibody titer against a particular virus [37]. However, our data showed that the use of pooled serum may mask the difference between two viruses. Hence, it is important to test and compare the titer of individual serum specimens.

There are several limitations in this study. First, since all the serum comes from individuals in Hong Kong, this may not reflect the situation in other places. For example, in some parts of Europe, A(H3N2) was the predominant subtype in the 2018/2019 winter influenza season. Second, we have used a limited number of influenza virus strains. Third, one mutation arose during the virus passage for H3N2-inter. However, for this mutation, the MN titer was the same against T160 or K160 strain when ferrets were infected with a natural strain (T160) of H3N2 [38]. Since most of the Hong Kong population has not been vaccinated, this should not affect our results substantially.

In summary, our results have demonstrated significant antigenic changes in the interepidemic emerging A(H1N1) virus, which was the virus subtype that predominated the 2018/2019 influenza season in Hong Kong. Our results support the use of human serum panels and MN assay in determining antigenic changes which are relevant to the human population, but further studies are required to assess whether this method is generalisable.

## Supplementary material

For supplementary material accompanying this paper visit https://doi.org/10.1017/S0950268819001717. click here to view supplementary material
